# Identification of Autophagy- and Ferroptosis-Related lncRNAs Functioned through Immune-Related Pathways in Head and Neck Squamous Carcinoma

**DOI:** 10.3390/life11080835

**Published:** 2021-08-16

**Authors:** Qi Guo, Xuehan Zhang, Tao Shen, Xiangting Wang

**Affiliations:** 1 Department of Geriatrics, Gerontology Institute of Anhui Province, The First Affiliated Hospital, Division of Life Sciences and Medicine, University of Science and Technology of China, Hefei 230026, China; gq0603@mail.ustc.edu.cn (Q.G.); zhangxh6@mail.ustc.edu.cn (X.Z.); 2 Anhui Provincial Key Laboratory of Tumor Immunotherapy and Nutrition Therapy, Hefei 230026, China; 3 Division of Life Sciences and Medicine, School of Life Sciences, University of Science and Technology of China, Hefei 230026, China; 4 Hefei National Laboratory for Physical Sciences at the Microscale, University of Science and Technology of China, Hefei 230026, China

**Keywords:** autophagy, ferroptosis, long non-coding RNA, head and neck squamous carcinoma, risk model, immune cell infiltration

## Abstract

The interplay between autophagy and ferroptosis has been highlighted as an important event to decide cancer cell fate. However, the underlying mechanisms remain largely unclear. In this study, we systematically explored the expression, prognostic value and functional roles of lncRNA in autophagy and ferroptosis. By a set of bioinformatics analyses, we identified 363 autophagy- and ferroptosis-related lncRNAs (AF-lncRNAs) and found 17 of them are dramatically related to the prognosis of head and neck squamous cell carcinoma (HNSC) patients, named as prognosis-related AF-lncRNAs (PAF-lncRNAs). Based on six key PAF-lncRNAs, a risk score model was developed and used to categorize the TCGA-retrieved HNSC patients into two groups (high-risk vs. low-risk). Functional analysis showed the differentially expressed genes (DEGs) between the two groups were mainly enriched in immune-related pathways and regulated by a PAF-lncRNA-directed ceRNA (competitive endogenous RNA) network. Combined with a variety of immune infiltration analyses, we also found a decreased landscape of immune cell infiltration in high-risk groups. Together, by revealing PAF-lncRNAs with tumor prognostic features functioned through immune-related pathways, our work would contribute to show the pathogenesis of a lncRNA-directed interplay among autophagy, ferroptosis and tumor immunity in HNSC and to develop potential prognostic biomarkers and targets for tumor immunotherapy.

## 1. Introduction

Autophagy-dependent cell death was described as a form of regulated cell death (RCD) that mechanistically depends on the autophagic machinery or components [1]. An increasing number of discoveries have built strong links between autophagy and various types of RCD, including ferroptosis [2,3,4]. Ferroptosis is an iron-dependent form of regulated cell death, which mainly depends on the accumulation of iron and reactive oxygen species (ROS) [5]. Studies have found that autophagic machinery could contribute to ferroptosis by mediating the degradation of ferritin and some genes that are involved in the crosstalk of ferroptosis and autophagy, and thus contribute to ferroptotic cancer cell death [4,6]. Thus far, emerging studies have implicated that the interplay between autophagy and ferroptosis decides cancer cell fate by activating integrated signaling pathways and influencing gene expression programs [3]. However, the underlying molecular mechanisms are still largely unclear.

Head and neck squamous cell carcinoma (HNSC) is a frequent malignancy worldwide with an incidence rate of approximately 900,000 new cases and half a million deaths annually [7]. Most patients are diagnosed at an advanced stage of HNSC, which is usually associated with a poor prognosis [8]. Although multiple treatment options are available, such as surgery, radiotherapy, and chemotherapy, the clinical heterogeneity and lack of early detection of HNSC cause the 5-year survival rate to be less than 50% [9]. Studies have shown that autophagy and ferroptosis are fundamental cellular events, which have been found to affect a variety of characteristics of HNSC, including proliferation, migration, and drug resistance [10,11,12,13]. While autophagy occurred in tumor cells, the formation of double-membrane autophagic vesicles could be observed by using a transmission electron microscope (TEM) [14]. While ferroptosis occurred in tumor cells, mitochondria were observed with decreased size, increased mitochondrial membrane densities, reduction or vanishing of mitochondria crista, and outer mitochondrial membrane under the scope of TEM [15,16,17]. In recent years, immunotherapy and targeted therapy have been incorporated into HNSC treatment and have become promising therapeutic options [18,19]. Increasing evidence revealed that autophagy and ferroptosis are critically involved in the regulation of anti-tumor immunity and might provide potential strategies in immunotherapy [20,21]. However, the connection between the HNSC immune landscape, autophagy and ferroptosis have not been elucidated.

Long non-coding RNAs (lncRNAs) represent a group of regulatory RNAs that are larger than 200 nucleotides. Increasing evidence has shown that lncRNAs are important regulators in almost all the physical and pathological events, including the occurrence and development of HNSC [22,23]. For example, our previous work showed that lncRNA 7SK could promote tongue squamous cell carcinoma’s RCD and thus act as an anti-tumor factor [24]. Recent studies have found that lncRNAs are involved in regulating autophagy through activating autophagy-related enzymes and shared clinical relevance with ferroptosis [25,26]. The key roles of lncRNAs as regulators of the immune response in cancer have progressively emerged [27]. However, it remains largely unclear about the comprehensive picture of lncRNAs’ engagement in autophagy, ferroptosis, and tumor immunity.

In the present study, we focused on investigating the engagement of lncRNAs in autophagy and ferroptosis. We identified 363 both autophagy- and ferroptosis-related lncRNAs (AF-lncRNAs), and six of them, namely key prognosis-related AF-lncRNAs (PAF-lncRNAs), have exhibited superior prognostic value for HNSC patients. A risk model based on these PAF-lncRNAs was further developed and used to divide the samples into two groups (high-risk vs. low-risk). Functionally, we found that the differentially expressed genes (DEGs) between high- and low-risk groups were dramatically enriched in immune-related pathways and regulated by a PAF-lncRNA-directed ceRNA network. Furthermore, the low-risk group patients were marked by high immune infiltration levels in the majority of the immune cell signatures, while the high-risk group patients were marked by low immune infiltration levels in the majority of the immune cell signatures. Together, our results comprehensively unveiled the engagement of autophagy- and ferroptosis-related lncRNAs in shaping the landscape of the HNSC immunity.

## 2. Materials and Methods

**Figure 1:**
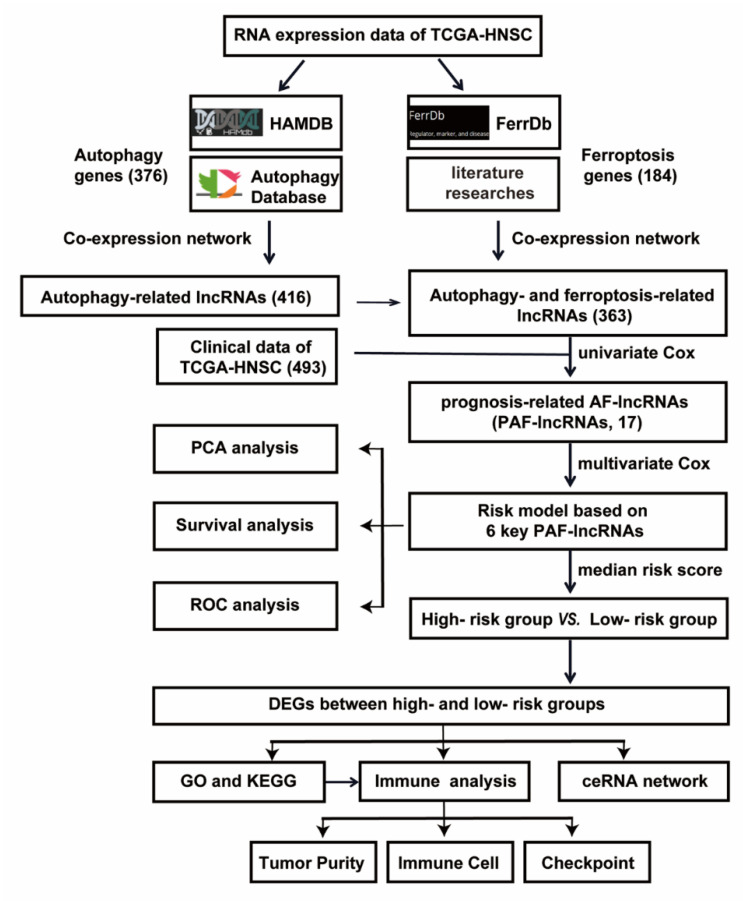
The flow-chart of data collection and analysis in HNSC.

The flowchart of data collection and analysis is shown in Figure 1. We elaborate on each step in the following sub-sections.

### 2.1. Data Resources

The RNA-sequence data (fragments per kilobase of exon model per million mapped, FPKM), clinical information and phenotype information of 527 HNSC patients were obtained from the XENA GDC database (http://xena.ucsc.edu/, accessed on 27 March 2020) [28]. We selected 493 tumors with complete follow-up information and survival time longer than 30 days. Phenotype and survival information of these patients are shown in Table S1. A total of 376 autophagic genes were gathered from the overlap of the Autophagy database (http://tp-apg.genes.nig.ac.jp/autophagy, accessed on 22 March 2021) [29] and Human Autophagy Modulator database (HAMdb, http://hamdb.scbdd.com, accessed on 22 March 2021) (Table S2) [30]. A total of 184 human ferroptotic genes were obtained from the FerrDb database (www.zhounan.org/ferrdb, accessed on 19 March 2021) [31] and literature studies (Table S2) [32,33].

### 2.2. Identification of Prognostic Autophagy- and Ferroptosis-Related LncRNAs

Firstly, 416 autophagy-related lncRNAs were identified by Pearson correlation analysis within mRNA and lncRNA expression according to the criteria of |Correlation Coefficient| > 0.4 and *p*-value < 0.05 (limma R package, Version:3.46.0) [34]. Second, we screened ferroptosis-related lncRNAs based on autophagy-related lncRNAs by using the same method. Then, we defined both autophagy-related lncRNAs and ferroptosis-related lncRNAs as autophagy- and ferroptosis-related lncRNAs (AF-lncRNAs).

In order to construct an AF lncRNA prognostic model, 493 patients were divided into a training set (60%, 294 samples) and testing set (40%, 199 samples). Univariate Cox proportional hazards analysis was used to identify prognosis-related AF-lncRNAs in the training set. Subsequently, multivariate Cox analysis was used to construct the prognostic risk model by employing the survival R package.

### 2.3. Development and Assessment of the Prognostic Risk Model

According to multivariate Cox analysis, the risk score was calculated by the following formula: risk score = coef (lncRNA1) × exp (lncRNA1) + coef (lncRNA2) × exp (lncRNA2) + … + coef (lncRNAn) × exp (lncRNAn) [35,36,37]. The coefficient values (coef) were calculated following previously reported methods [37]. The coef represents the coefficient of the corresponding lncRNA, which was calculated by using the survival coxph function of the R package. The “exp” represents the expression of corresponding lncRNA. Based on the median risk-score, HNSC patients in the training set and testing set were divided into high- and low-risk groups, respectively. The univariate and multivariate Cox regression analyses were used to calculate the prognostic value of risk-score and clinical features by survival R package. The landscape of survival status was described between the high- and low-risk patients by using the ggplot2 and pheatmap R packages. The principal component analysis (PCA) was applied to verify the classification between high- and low-risk groups. Then Kaplan–Meier survival analysis was performed to estimate the survival difference between these two groups by using the survival and survminer R packages. In order to estimate the sensitivity and specificity of PAF-lncRNAs signatures, we employed time-dependent receiver operating characteristic (ROC) curves and multi-factor ROC curves by using the timeROC (Version:0.4) and survivalROC R package (Version:1.0.3).

### 2.4. Functional Analysis of the DEGs between High- and Low-Risk Groups

The differentially expressed genes between high- and low-risk groups were screened out by the limma R package with the criteria of |log2 (fold change)| > 1 and *p* < 0.05. The Gene Ontology (GO) and Kyoto Encyclopedia of Genes and Genomes (KEGG) were performed by limma and clusterProfiler R packages. We used GOplot and ggplot2 R packages to display the result of the functional enrichment analysis.

### 2.5. Estimation of Immune Cell Infiltration Proportion

An estimation algorithm was used to calculate the stromal score, immune score, and estimate score by estimate and utils R packages [38]. ImmuCellAI and CIBERSORT algorithms were used to estimate the proportion of immune cell infiltration, respectively. For ImmuCellAI, the estimated immune cell proportion of 24 types were calculated by the Immune Cell Abundance Identifier website (ImmuCellAI) [39]. For the CIBERSORT algorithm, the 22 human hematopoietic cell phenotypes file (LM22.txt) was obtained and the immune cell proportion of 22 types were estimated by performing “source (“CIBERSORT.R”)” [40].

### 2.6. Network of ceRNAs and lncRNA-RPB

The interactions of miRNA, PAF-lncRNAs and DEGs were collected from ENCORI (http://starbase.sysu.edu.cn/index.php, accessed on 13 May 2021) [41]. The interactions between PAF-lncRNAs and RBP were predicted in the RNAct database (https://rnact.crg.eu/, accessed on 1 June 2021) [42] and collected from ENCORI. Networks were constructed and visualized by using Cytoscape software (version 3.8.0) [43].

### 2.7. Statistical Analysis

All statistical analyses were performed by using R software (version 4.0.3). For each analysis, statistical significance was set at *p* < 0.05 without special description. Non-normally distributed variables were analyzed using the Wilcoxon test, and the Benjamin Hochberg method was used to calculate FDR. The survival curves between high- and low-risk groups were assessed by using the Kaplan–Meier survival analysis with the log-rank test.

## 3. Results

### 3.1. Identification of Autophagy- and Ferroptosis-Related lncRNAs (AF-lncRNAs) in HNSC

**Figure 2:**
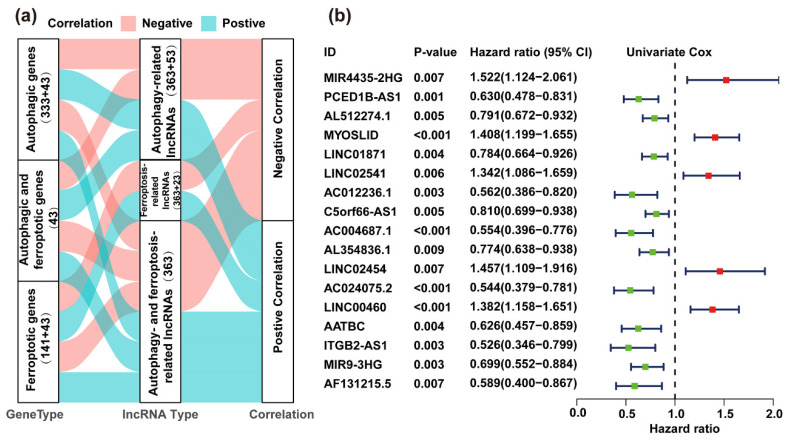
Identification of the prognosis-related AF-lncRNAs (PAF-lncRNAs). (**a**) Sankey diagram shows the correlation of lncRNA-mRNA between autophagy and ferroptosis by co-expression correlation analysis. (**b**) Forest diagram shows the HR (95% CI) and *p*-value of 17 PAF-lncRNAs by univariate Cox proportional hazards analysis.

To identify the autophagy and ferroptosis correlated lncRNAs in HNSC, we used autophagic and ferroptotic genes to construct co-expression networks in HNSC patients according to the criteria of |Correlation Coefficient| > 0.4 and *p* < 0.05 by Pearson correlation analysis. Our results revealed 363 autophagy- and ferroptosis-related lncRNAs, namely, AF-lncRNAs (Figure 2a, Table S2).

### 3.2. Construction of a Prognostic Risk Model in the TCGA Cohort Based on the Identified Prognostic AF-lncRNAs in HNSC

**Figure 3:**
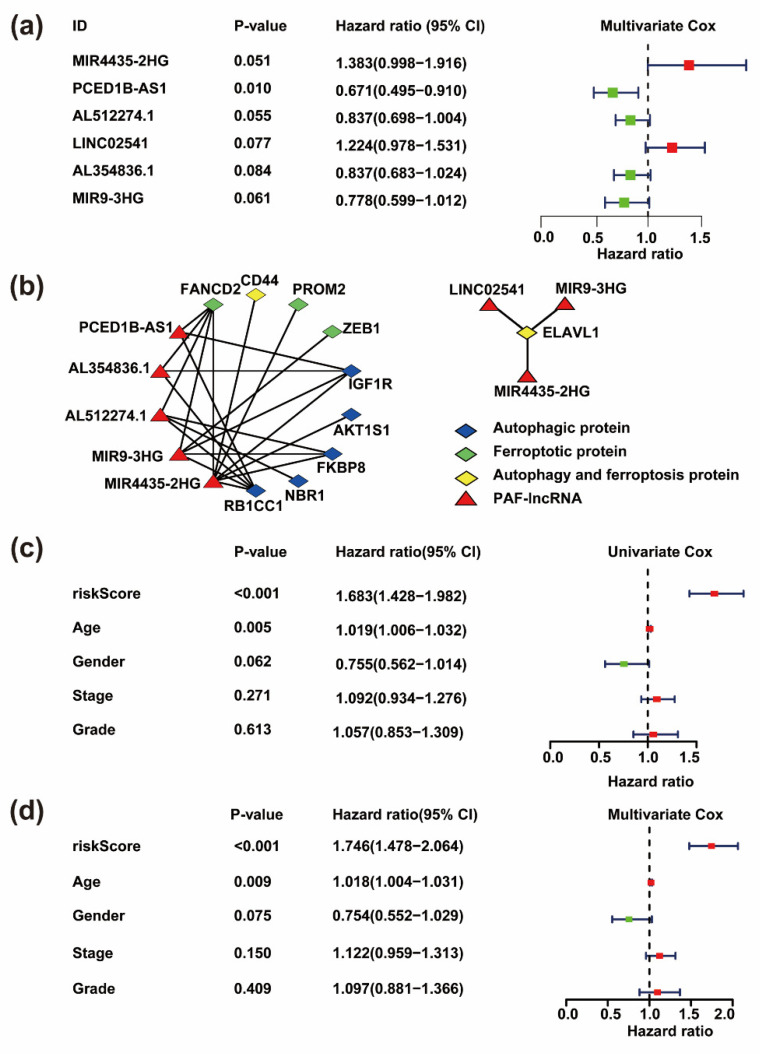
Development and assessment of prognostic risk model based on six key PAF-lncRNAs in HNSC. (**a**) Forest diagram shows the HR (95% CI) and *p*-value of six key PAF-lncRNAs in a risk model derived from multivariate Cox proportional hazards analysis. (**b**) LncRNA-protein network between PAF-lncRNA, autophagic and/or ferroptotic proteins. Left: the lncRNA-protein networks collected from RNAct database. Right: the lncRNA-protein networks collected from ENCORI database (http://starbase.sysu.edu.cn/index.php; accessed on 13th May 2021). Edges: the associations of lncRNA-protein; red nodes: PAF-lncRNAs; blue nodes: autophagic proteins; green nodes: ferroptotic proteins; yellow nodes: autophagic and ferroptotic proteins. (**c**,**d**) Independent prognostic analysis of risk model and clinical features by univariate Cox analysis and multivariate Cox analysis.

To investigate the prognostic value of AF-lncRNAs in HNSC patients, we performed univariate Cox analysis to estimate the prognostic relationship between AF-lncRNAs and overall survival (OS) in 493 HNSC samples from TCGA. Our result showed that 17 lncRNAs, referred to as prognostic AF-lncRNAs (PAF-lncRNAs), were significantly associated with the survival of HNSC patients (*p* < 0.01, Figure 2b, Table S3). We subsequently performed the multiple stepwise cox regression analysis to investigate the impact of these 17 prognostic-associated AF-lncRNAs on patient survival time and clinical outcomes. *PCED1B-AS1*, *AL512274.1*, *AL354836.1*, *MIR9-3HG*, *MIR4435-2HG* and *LINC02541* were found to be independent factors in HNSC (Figure 3a, Table S3). Then, we identified a network between the six key PAF-lncRNAs and autophagy and/or ferroptosis genes. Most of these genes, including *CDD4*, *ELAVL1*, *FKBP8*, *NBR1*, *RB1CC1*, and *ZEB1*, were correlated with patients’ overall survival, which again supports that the PAF-lncRNAs were correlated with the process of autophagy and/or ferroptosis (Figure 3b and Figure S1).

Utilizing the aforementioned independent PAF-lncRNAs, we next constructed a prognostic predictive model. The risk-score of each patient was calculated according to the following formula (Table S4):

Risk Score = (0.3242 × Exp*MIR4435-2HG*) + (−0.3983×Exp*PCED1B-AS1*) + (−0.1779 × Exp*AL512274.1*) +     (0.2019 × Exp*LINC02541*) + (−0.1783 × Exp*AL354836.1*) + (−0.2507 × Exp*MIR9-3HG*).

By using univariate and multivariate Cox regression analysis, we compared the prognostic significance of risk-score and different clinical characteristics in HNSC. Our results showed that only age and risk-score calculated by the six PAF-lncRNAs were dramatically correlated with HNSC OS, and the risk-score was shown to be the most significant parameter (*p* < 0.05, Figure 3c,d). Together, these data suggested the intimated connection of six PAF-lncRNAs with autophagy and ferroptosis, and their relationship is associated with HNSC prognosis.

### 3.3. The Risk Model Exhibited Robust Predictive and Discriminative Ability for HNSC Patients

**Figure 4:**
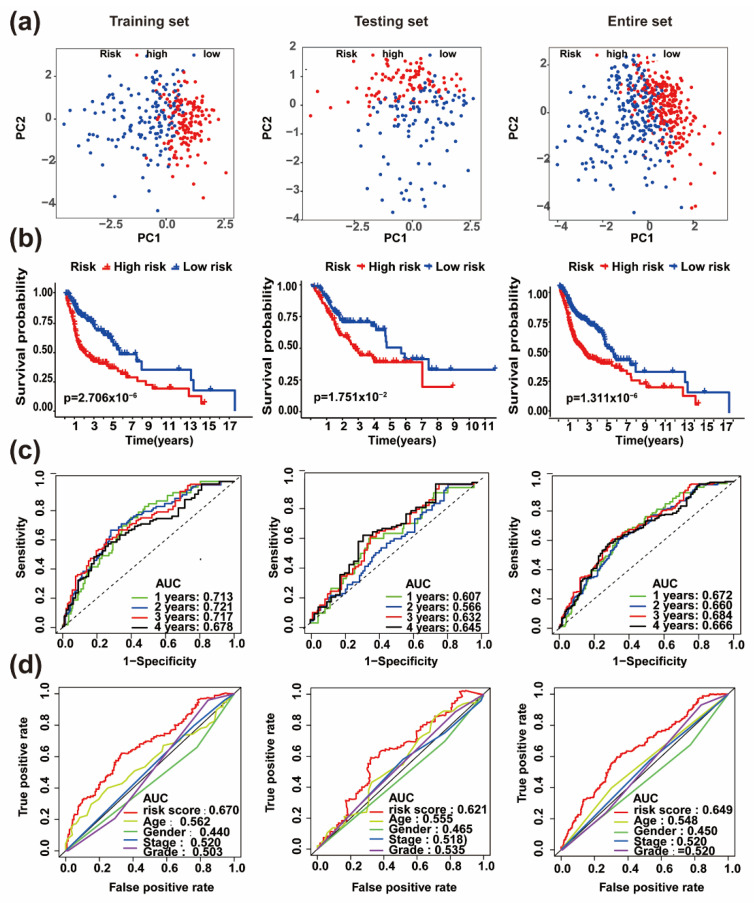
Validation of the prognostic risk model in HNSC patients. (**a**) The principal component analysis (PCA) shows the distinguished distribution of high- and low-risk patients based on the risk model. (**b**) The overall survival of the high- and low-risk patients in the indicated training, testing, and entire sets. (**c**) Time-dependent ROC curves (1, 2, 3, 4 years) analysis for survival prediction verified the prognostic performance of the risk-score model in the indicated training, testing, and entire sets. (**d**) The ROC curves exhibit superior performance of risk-score compared to other measured characteristics in the indicated training, testing, and entire sets.

Based on the risk model, HNSC patients from TCGA were divided into high- and low-risk groups by calculating median risk-score. The PAF-lncRNAs expressions, survival status and risk-scores of these patients were displayed in Figure S2. To assess the accuracy of the stratification, we conducted a set of bioinformatic analyses. First, principal component analysis (PCA) results suggested that risk-score works well in distinguishing high-risk patients with low-risk groups in the TCGA training, testing, and entire sets (Figure 4a). Second, Kaplan–Meier analyses showed the patients’ OS rate was dramatically lower in the high-risk group compared to patients in the low-risk group in the TCGA training, testing, and entire sets (Figure 4b). Third, time-dependent receiver operating characteristic (ROC) curves were calculated, and the area under the ROC curves (AUC) of risk-scores at 1, 2, 3 and 4 years for survival prediction were all above 0.6, which suggested the risk-score had moderate prediction accuracy in TCGA training, testing, and entire sets (Figure 4c). In addition, the AUC of the risk-score exhibited moderate performance compared to other measured phenotypes in predicting the prognosis of HNSC patients in TCGA training, testing, and entire sets (Figure 4d). Taken together, these results suggested that the risk model based on the six PAF-lncRNAs has good predictive ability and stratification accuracy for HNSC patients.

### 3.4. Differentially Expressed Genes (DEGs) between High- and Low-Risk Groups Were Dramatically Enriched in Immune-Related Pathways

**Figure 5:**
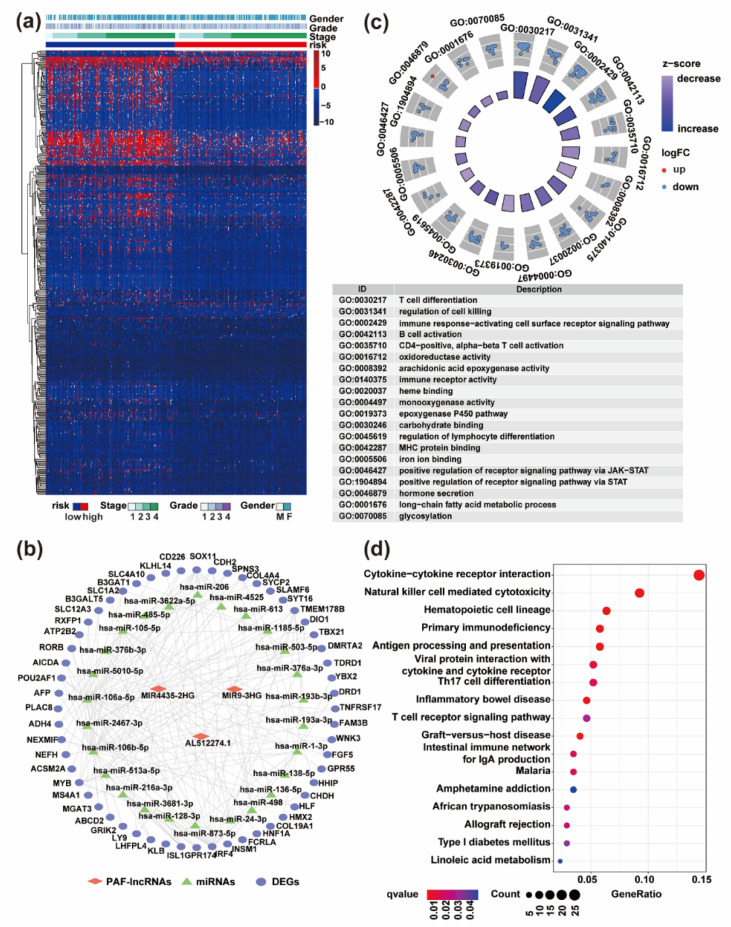
Functional enrichment of differentially expressed genes (DEGs) between high- and low-risk groups. (**a**) Heatmap shows the expression data of DEGs and the clinicopathological manifestations in high- and low-risk groups. (**b**) The ceRNA network between AF-lncRNAs, miRNAs, and DEGs. (**c**) Functional enrichment of the DEGs in high- and low-risk groups by GO analysis (*p* < 0.05). (**d**) Pathway enrichment of the DEGs in high- and low-risk groups by KEGG analysis (*p* < 0.05).

To study the differences between high- and low-risk groups at the whole genome-wide level, we performed differential expression analysis. A total of 437 DEGs (down-regulation: 411 and up-regulation: 26) between two groups were identified (Table S5), and the expressions of them were displayed in a heat map (Figure 5a). We also constructed a PAF-lncRNAs ceRNA network through the ENCORI database to explore the interaction between lncRNAs and DEGs (Figure 5b). We found that there are 3 PAF-lncRNAs, *AL512274.1*, *MIR4435-2HG*, and *MIR9-3HG*, which might regulate the 56 DEGs through 26 miRNAs. The majority of the DEGs, e.g., *SOX11*, *TBX21*, *FAM3B*, *FGF5*, *HNF1A*, *MYB*, and *PLAC8* have been reported to function as promoters or biomarkers in various cancer types [44,45,46,47,48,49,50]. Among these, PLAC8 were found to regulate autophagy-related functions in a variety of cancers [51,52]. In addition, numerous DEGs were reported to be involved in the proliferation and differentiation of immune cells and could regulate immune functions, including *CD226*, *IRF4*, *LY9*, *MS4A1*, *TBX21*, *TNFRSF17*, *FCRLA*, and *SLAMF6* [45,53,54,55,56,57,58].

To further characterize and understand the biological insights of these DEGs, we performed gene ontology (GO) and KEGG analysis. The DEGs were mainly enriched in immune-related processes, such as T cell differentiation, B cell activation, immune receptor activity, cytokine receptor binding, regulation of lymphocyte and CD4-positive, alpha-beta T cell activation (Figure 5c). KEGG also displayed that the DEGs were related to immune pathways (Figure 5d). Together, these results indicated PAF-lncRNAs might mediate the generation of immune microenvironmental differences of HNSC high- and low-risk groups.

### 3.5. Distinct Immune Landscapes between High- and Low-Risk HNSC Patients Were Identified

**Figure 6:**
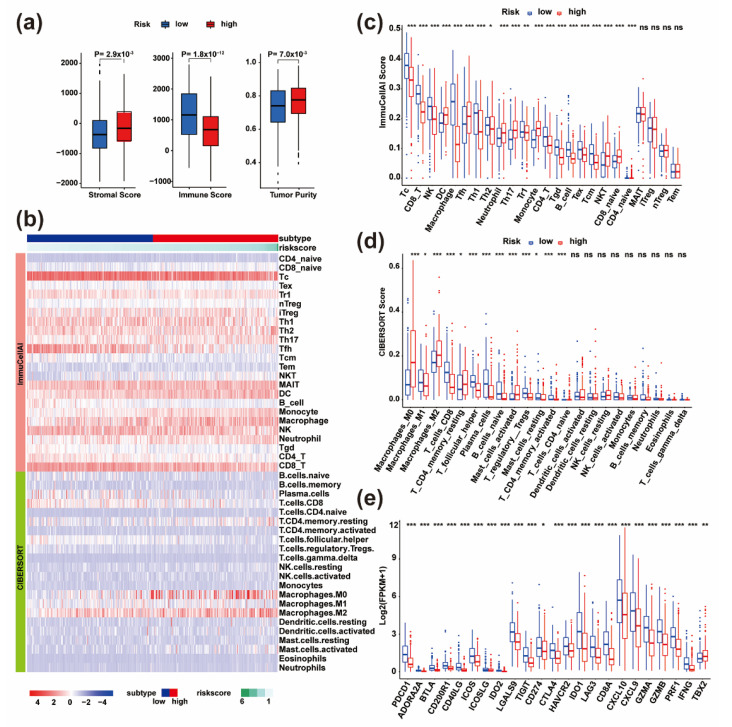
Comprehensive analyses of immune microenvironment and immune checkpoints between high- and low-risk groups. (**a**) The boxplot shows the immune infiltration and tumor purity between high- and low-risk groups. (**b**) The heat map shows the proportions of various types of immune cell infiltration in each HNSC patient by two algorithms. (**c**) The difference of immune cell infiltration proportions between high- and low-risk groups from the ImmuCellAI algorithm. (**d**) The difference of immune cell infiltration proportions between high- and low-risk groups by the CIBERSORT algorithm. (**e**) The differential expression of immune checkpoints and immune activation signals between high- and low-risk groups. * *p* < 0.05, ** *p* < 0.01, *** *p* < 0.001.

Following the aforementioned results, we then systematically investigated the immune landscape of the two risk groups. Firstly, we compared the tumor immune microenvironment between high- and low-risk groups (Figure 6a, Table S6). Our data showed that the high-risk group was marked by higher tumor purity and lower immune infiltration levels than the low-risk group (Figure 6b). Secondly, we compared the immune cell composition of the tumor immune microenvironment by using the ImmuCellAI and CIBERSORT algorithm, respectively (Figure 6b). According to the ImmuCellAI calculation results, the largest proportion of infiltrating immune cells in HNSC patients were cytotoxic T cells (Tc), CD4 + T cells, CD8 + T cells, NK cells, Tfh and Th1 (Figure 6C, Table S7). The relative proportion of these immune cells was significantly higher in low-risk patients than in high-risk patients (*p* < 0.05; Wilcoxon test, Figure 6c). However, the proportions of dendritic cells, monocytes, macrophages and neutrophils in the high-risk group were significantly higher than the low-risk group (*p* < 0.05; Wilcoxon test, Figure 6c). Specifically, the CIBERSORT algorithm can distinguish the subtypes of macrophages. By using this algorithm, we found a higher proportion of the M2 macrophages in the high-risk group, while M1 macrophages were higher in the low-risk group (Figure 6d, Table S7). As reported in previous studies, macrophages undergo a switch that leads to differentiation into either inflammatory (M1) or regulatory (M2) subtypes. Among these, M1 is mainly involved in tumor killing, while M2 is mainly involved in supporting tumor growth [59]. Our results suggested that the polarization of macrophages might be a regulatory mechanism for the difference of survival between the high- and the low-risk group. Thirdly, the two groups showed significant differences in immune checkpoint and immune activation gene expression levels (*p* < 0.05; Figure 6e, Table S8). The high-risk group was associated with relatively lower immune checkpoint and activation signal expression levels, whereas the low-risk group was associated with the higher expression level.

In summary, our results revealed that immune-driven cells are associated with the low-risk group, while immune-regulatory cells tend to be more common in the high-risk group. The consistency between the immune profile and prognostic profile in the two groups also implied that our classification method is scientific and reasonable. As previous studies have shown that the abundance of T cell subsets, particularly that of tumor infiltrating T cells, could influence clinical curative effects and prognosis [60,61], our risk model has the potential to be applied for predicting the immunotherapy response.

## 4. Discussion

As two closely linked forms of RCD, increasing evidence has shown that autophagy and ferroptosis are intimately associated with tumor progression [62,63,64]. However, the engagement of lncRNAs in HNSC autophagic and ferroptotic processes have not been thoroughly and systematically studied. In the present study, we systematically investigated the expression and prognostic relevance of 363 autophagy- and ferroptosis-related lncRNAs (AF-lncRNAs) in HNSC. Previous reports have shown the connection between lncRNAs and RCD, and their relationship is associated with tumor progression [65,66,67]. In this paper, by performing univariate cox regression analysis, multiple stepwise cox regression analysis, survival analyses and ROC analyses, etc., a total of six key PAF-lncRNAs in HNSC were identified, including *MIR4435-2HG*, *PCED1B-AS1*, *AL512274.1*, *LINC02541*, *AL354836.1*, and *MIR9-3HG*. Previous studies have reported that the expression of *MIR4435-2HG*, *PCED1B-AS1*, *AL512274.1*, *AL354836.1*, *MIR9-3HG* were associated with tumorigenesis and regulated cell death in various tumor types, which support the further identification and exploration in HNSC [68,69,70,71,72]. For example, overexpression of *MIR4435-2HG* will promote tumor cell proliferation while knockdown of *MIR4435-2HG* will lead to cell death [73,74,75,76]. In addition, researchers have found that inhibition of *MIR4435-2HG* would downregulate *Nrf2*, which would alter the resistant status of head and neck cancer cells to a more sensitive status to ferroptosis and eventually promote ferroptotic cell death [13,74].

Functional analyses revealed that these PAF-lncRNAs have a close relationship with several autophagic and/or ferroptotic genes, including *FANCD2*, *CD44*, *PROM2*, *ZEB1*, *IGF1R*, *AKT1S1*, *FKBP8*, *NBR1*, *RB1CC1*, and *ELAVL1*. Among these, *IGF1R*, *AKT1S1*, *FKBP8*, *NBR1*, and *RB1CC1* have been reported to function as regulatory factors, such as autophagic adaptors or receptors, to regulate the autophagic processes [77,78,79,80,81]. *FANCD2*, *PROM2*, and *ZEB1* have been reported to regulate ferroptosis through regulating the accumulation of lipid ROS and intracellular iron export, etc. [82,83,84]. As for *ELAVL1* and *CD44*, they have been reported to regulate the interplay of autophagic and ferroptotic processes [85,86,87]. For example, *ELAVL1* could promote ferroptosis by regulating autophagy in myocardial ischemia/reperfusion injury [85]. Autophagic flux impairment induced a high expression of *CD44* and thus induced mitochondrial dysfunction, oxidative stress and cancer cell death [86]. The interaction between PAF-lncRNAs and these autophagic and/or ferroptotic genes indicated that PAF-lncRNAs might participate in the regulation of autophagy and ferroptosis and thus mediate autophagic and ferroptotic tumor cell death through these genes. However, its detailed molecular mechanism still needs further future genetic and experimental studies.

We then produced a risk model based on these PAF-lncRNAs. The ROC analysis revealed that these PAF-lncRNAs signatures have better diagnostic capability of selecting the high-risk HNSC patients with poor prognosis. Based on the risk model, 493 HNSC patients from TCGA were divided into high- and low-risk groups. The divided high- and low-risk patients showed distinct gene expression patterns, and the DEGs were dramatically enriched in many immune-related pathways.

Tumor immunity and RCD have been linked together from recent reports [88,89]. Although several findings have supported the importance of immunology in HNSC [90,91,92], the underlying molecular mechanism and potential modulation between tumor immunity and RCD, especially autophagy and ferroptosis, are largely unclear. In this paper, GO and KEGG analyses based on the DEGs enriched many immune-related biological processes and pathways. For instance, T cell differentiation is the process in which precursor cell types acquire characteristics of more mature T cells to achieve immune effects, while B cell activation is defined by the change in morphology and behavior of mature or immature B cells. The function of immune receptor activity is to receive signals and transmit them into cells to initiate the immune response. In addition, we established a connection between 56 DEGs and PAF-lncRNAs from the ENCORI database, which was based on the CLIP-seq data. Numerous independent studies have validated the regulatory roles of the six PAF-lncRNA on those DEGs through competitive binding of the miRNA regulators. For example, PCED1B-AS1 could inhibit tumor cell death by cooperating with the *miR-194-5p/PCED1B* axis in glioma [93], and *MIR4435-2HG* could regulate cancer proliferation through sponging *miR-206*, *miR-802*, *miR-128-3 and miR-1224-5p* and regulated *YAP1*, *FLOT2*, et al., in various cancers [73,94,95,96]. Among all these DEGs, nearly half (24/56) have been reported to be involved in activation and differentiation of immune cells or regulation of the immune response [45,53,54,55,56,57,58]. One of them, placenta associated 8 (*PLAC8*), has been reported to regulate autophagy by suppressing the production of IL-18, which is a pro-inflammatory cytokine that is capable of stimulating interferon gamma production and of regulating T helper cell responses [97]. These pieces of evidence support the idea that the PAF-lncRNAs might participate in regulating HNSC through the tumor immunity process.

HNSC is a disease that was previously characterized by immunosuppression [98]. Recently, considerable progress has been made in immune checkpoint inhibitor (ICI)-based HNSC treatment. However, the response rate of recurrent or metastatic HNSC to PD-1/PD-L1 inhibitors is only 13.3–22%, as per previous clinical trials [98]. Therefore, the selection of patients that can effectively respond to ICIs is crucial. For this reason, we estimated the immune cell infiltration of HNSC tumor samples, and results revealed the discriminated immune microenvironment landscapes of distinct risk groups. The infiltration ratio of effective T cells, NK cells, T helper cells, B cells and M1 macrophages that were related to anti-tumor effects were higher in the low-risk group. Effective T cells and B cells play critical roles in tumor control, and T helper cells can stimulate B cells for an immune response. The natural killer (NK) cells were discovered for their ability to recognize and kill tumor cells [99] and to release a number of cytokines that regulate both innate and adaptive immune responses [100]. As for M2 macrophages, the proportions of dendritic cells (DCs), mast cells and neutrophils that related to pro-tumor effects were significantly higher in the high-risk group than low-risk group by both algorithms. Due to the complex phenotype and cancer heterogeneity, the infiltration of dendritic cells and mast cells have controversial results in predicting clinical outcomes in different tumors [101,102,103]. Because parameters of the immune contexture have been associated with treatment efficacy, it is important to characterize the baseline HNSC immune milieu to clarify the composition and property of tumor-infiltrating immune cells [104]. Together, these results supported the involvement of PAF-lncRNAs in regulating HNSC tumor immunity. Thus, the identification of PAF-lncRNAs not only provides a potential target for anti-cancer immunotherapy but also build a bridge between RCD and immunogenicity of HNSC, which might shed new light on revealing another layer of lncRNA-directed immunogenicity of cancer cells.

## 5. Conclusions

In summary, we systematically explored the expression and prognostic value of autophagy- and ferroptosis-related lncRNAs by a series of bioinformatics analyses in HNSC. Our study revealed six prognosis-related AF-lncRNAs and developed a novel prognostic model based on these lncRNAs. This model proved to be an independent prognostic factor, which has a favorable predictive effect on prognosis for HNSC. In addition, we revealed these AF-lncRNAs functioned through multiple critical tumor immune-related processes. Our results would contribute to show the pathogenesis of HNSC and to develop new treatment targets and prognostic molecular markers.

## Data Availability

The datasets supporting the conclusions of this article are included within the article and its additional files. The datasets analyzed during the current study are available in the XENA GDC database (http://xena.ucsc.edu/, accessed on 27 March 2020), Autophagy Database (http://tp-apg.genes.nig.ac.jp/autophagy, accessed on 22 March 2021), Human Autophagy Modulator Database (HAMdb, http://hamdb.scbdd.com, accessed on 22 March 2021), FerrDb database (www.zhounan.org/ferrdb, accessed on 19 March 2021), ENCORI (http://starbase.sysu.edu.cn/index.php, accessed on 13 May 2021), and RNAct database (https://rnact.crg.eu/, accessed on 1 June 2021).

## Supplementary Materials

The following are available online at https://www.mdpi.com/article/10.3390/life11080835/s1, Figure S1: Expression heat map, risk score distribution, and survival status of TCGA-retrieved HNSC patients, Table S1: Clinical characteristics of TCGA-retrieved HNSC patients in the indicated sets, Table S2: The list of autophagic genes, ferroptotic genes, and AF-lncRNAs, Table S3: Univariate /Multivariate Cox proportional hazards analysis, Table S4: List of survival time, status, and risk-score of TCGA-retrieved HNSC patients, Table S5: DEGs between high- and low-risk patients, Table S6: List of stromal score, immune score, estimate score and tumor purity in the indicated groups, Table S7: List of ImmuCellAI score, CIBERSORT score in the indicated groups, Table S8: The expression of immune check point genes in the indicated groups.

## Abbreviations

AF-lncRNAs	autophagy- and ferroptosis-related lncRNAs
HNSC	head and neck squamous cell carcinoma
PAF-lncRNAs	prognosis-related AF-lncRNAs
DEGs	differentially expressed genes
RCD	regulated cell death
ROS	reactive oxygen species
OS	overall survival
lncRNAs	long non-coding RNAs
FPKM	fragments per kilobase of exon model per million mapped
PCA	principal component analysis; ROC, receiver operating characteristic
GO	gene ontology
KEGG	Kyoto Encyclopedia of Genes and Genomes
ImmuCellAI	immune cell abundance identifier
CIBERSORT	cell-type identification by estimating relative subsets of RNA transcripts
AUC	area under the ROC curve
miRNAs	microRNAs
