# Supplementary material for: Identification of Autophagy- and Ferroptosis-Related lncRNAs Functioned through Immune-Related Pathways in Head and Neck Squamous Carcinoma
Source: Life (Basel). 2021 Aug 16;11(8):835. doi: 10.3390/life11080835 (PMC8399325; doi:10.3390/life11080835)
Supplement: Supplementary file 1 [file life-11-00835-s001.zip › Revised Supplementary Figure_life-1314859.pdf]

Supplementary Figure

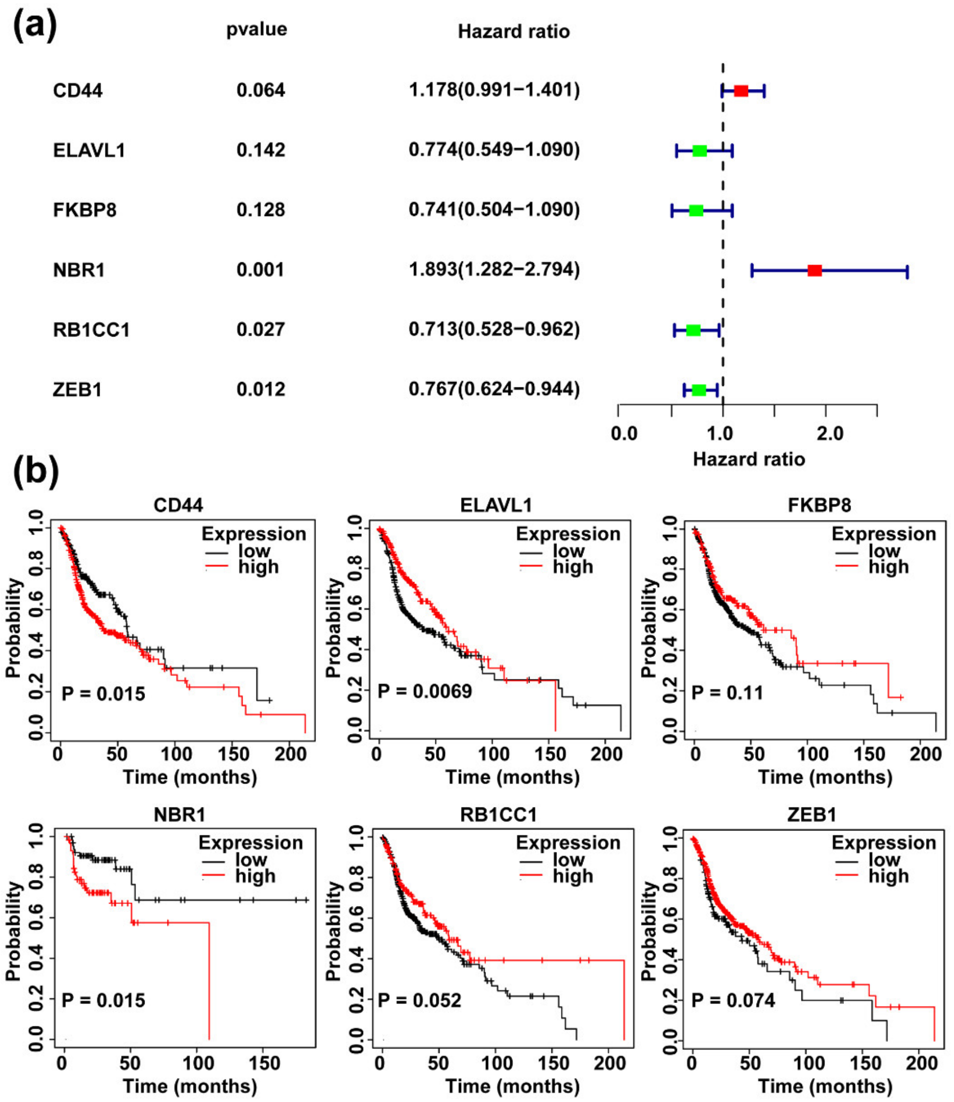

***S*upplementary Figure S1.** Multivariate Cox regression analysis and KM-plot analysis for the relationship of autophagic/ferroptotic proteins and overall survival. (a). Forest diagram shows the HR (95% CI) and P-value of autophagy/ferroptosis proteins by multivariate Cox proportional hazards analysis. (b). KM-plot shows the overall survival between the high- and low-expression of autophagic/ferroptotic genes.

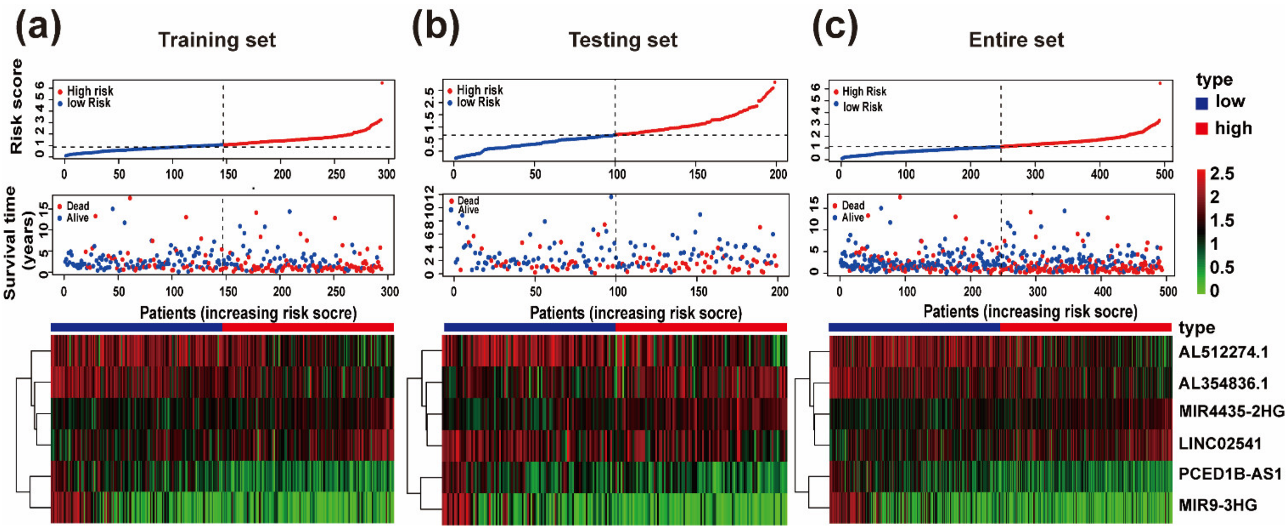

Supplementary Figure S2. Expression heat map, risk score distribution, and survival status of TCGA-retrieved HNSC patients. (a) The training set (n=278 tumor samples). (b) The testing set (n=183 tumor samples). (c) The entire set (n=461 tumor samples). Each group consists of three rows: top row, the risk-scores of each HNSC patients; middle row, the survival status and duration of each HNSC patients; bottom row, the expression of the 6 PAF-lncRNAs in HNSC patients. The color, from green to red shows, low to high expression.
